# ASO Author Reflections: Surgical Procedures for Early-Stage Lung Cancer Patients with Low Muscle Mass

**DOI:** 10.1245/s10434-024-16481-5

**Published:** 2024-11-15

**Authors:** Daisuke Ueda, Takahiro Mimae, Morihito Okada

**Affiliations:** Department of Surgical Oncology, Research Institute for Radiation Biology and Medicine, Hiroshima University, Hiroshima, Japan

## Past

A large prospective randomized trial demonstrated that segmentectomy was superior to lobectomy in terms of overall survival (OS) for patients with small-size non-small cell lung cancer.^1^ Sarcopenia and low muscle mass have become widely recognized risk factors for a poorer prognosis in malignant conditions. Although the authors’ group has demonstrated that low preoperative elector spinae muscle (ESM) mass is associated with poor postoperative OS,^2^ the impact of preoperative ESM mass and surgical procedure types on the postoperative prognosis remains unclear.

## Present

This study defined high or low ESM mass and investigated the impact of the index on prognosis according to surgical procedures. A review of 162 patients with lobectomy and 209 patients with segmentectomy showed that patients with low ESM mass had significantly poorer OS rates and higher non-lung-cancer-related mortality rates than those with high HA-ESMs in the lobectomy group, whereas no significant differences were found in the segmentectomy group. Low ESM mass was an independent prognostic factor for OS in the lobectomy group, but not in the segmentectomy group.^3^ One possible reason for this finding is the lower invasiveness of segmentectomy compared with lobectomy.

## Future

Patients with low muscle mass are more likely to benefit from segmentectomies than from lobectomies in terms of mortality due to other causes. The surgical procedure should be selected based on patients’ background including their muscle mass.
